# A prospective, randomized, single - blind study comparing intraplaque injection of thiocolchicine and verapamil in Peyronie's Disease: a pilot study

**DOI:** 10.1590/S1677-5538.IBJU.2015.0598

**Published:** 2016

**Authors:** I. L. Toscano, M.V. Rezende, L. F. Mello, L. Pires, D. Paulillo, S. Glina

**Affiliations:** 1 Departamento de Urologia, Hospital Ipiranga, São Paulo, Brasil

**Keywords:** Penile Induration, Penis, Verapamil, Colchicine

## Abstract

**Objectives:**

To compare the response to tiocolchicine and verapamil injection in the plaque of patients with Peyronie's disease.

**Materials and Methods:**

Prospective, single-blind, randomized study, selecting patients who have presented Peyronie's disease for less than 18 months. Thiocolchicine 4mg or verapamil 5mg were given in 7 injections (once a week). Patients who had received any treatment for Peyronie's disease in the past three months were excluded. The parameters used were the International Index of Erectile Function (IIEF-5) score, analysis of the curvature on pharmaco-induced erections and size of the plaque by ultrasonography.

**Results:**

Twenty-five patients were randomized, 13 received thiocolchicine and 12 were treated with verapamil. Both groups were statistically similar. The mean curvature was 46.7° and 36.2° before and after thiocolchicine, respectively (p=0.019) and 50.4° and 42.08° before and after verapamil, respectively (p=0.012). The curvature improved in 69% of patients treated with thiocolchicine and in 66% of those who received verapamil. Regarding sexual function, there was an increase in the IIEF-5 from 16.69 to 20.85 (p=0.23) in the thiocolchicine group. In the verapamil group the IIEF-5 score dropped from 17.50 to 16.25 (p=0.58). In the thiocolchicine group, the plaque was reduced in 61% of patients. In the verapamil group, 8% presented decreased plaque size. No adverse event was associated to thiocolchicine.

**Conclusion:**

The use of thiocolchicine in Peyronie's disease demonstrated improvement on penile curvature and reduction in plaque size. Thiocolchicine presented similar results to verapamil in curvature assessment. No significant side effects were observed with the use of tiocolchicine.

## INTRODUCTION

Peyronie's Disease (PD) occurs in about 8 to 9% of adult male Caucasians (1), and can be described as a localized alteration in the penile connective tissue involving the tunica albuginea of the corpus cavernosum. It is characterized by formation of an inelastic plaque that causes shortening of the tunica, resulting in penile deformity and pain (2, 3) during erection, thus jeopardizing penetration and sexual intercourse; moreover, it emotionally affects the patients and their partners (4).

Its pathogenesis remains poorly understood and several theories were proposed. Most specialists support the idea of a combination of microtrauma during sexual activity in a predisposed patient, which would lead to deregulation of the scarring process and formation of the plaque (5).

PD presents with two phases: the active phase, in which the penile deformity may vary with time and can last up to 18 months, and the stable phase, when the alteration of the penile form stabilizes (6).

Treatment for PD is individualized, according to the phase of the disease. During the active phase, noninvasive treatments are used and surgical correction is reserved to treat penile deformities during the stable phase of PD (7).

During the initial phase of PD, several treatments have been used, both orally and by intraplaque injection, with low success rates (5, 7).

The use of verapamil in the treatment of PD has gained prestige in medical literature since the publication by Lee and Ping, who described its action in increasing the activity of collagenase (1, 8). Many publications in the literature, both in laboratory animals (9) and human beings, confirmed the efficacy of verapamil in pain relief, in improved curvature (especially in non-calcified plaques with smaller curvatures, less than 30 degrees), with no significant adverse effects related to the injectable use of the drug.

Colchicine is an anti-inflammatory medication that has been widely used orally in rheumatologic diseases, such as gout (10, 11). More recently, it has been used in Peyronie's disease due to its capacity to inhibit mitoses of collagen-producing cells and to inhibit the fibroblast growth factor (10, 12, 13). The use of colchicine injected directly into the penile plaque during the acute phase of the disease could have greater efficacy than oral use, due to higher levels of the drug in the tissue.

However, parenteral use of colchicine may cause bone marrow depression, with severe pancytopenia and death (14, 15). Thus, thiocolchicine or thiocochicoside was chosen, which is a sulfur derivative of a natural glycoside of colchicine that acts as a muscle relaxant and has no side effects listed with colchicine. In this paper, intraplaque-injected thiocolchicine and verapamil were compared in patients with PD.

## MATERIALS AND METHODS

Twenty-five patients with Peyronie's disease, with up to 18-month progression, were randomized into two groups: one with 13 patients receiving a 2mL injection containing 4mg of thiocolchicine, and another with 12 patients receiving 2mL of a solution containing 5mg of verapamil. The solutions were injected using a 0.3×8mm needle in many punctures into the plaque.

The drugs were applied once a week, in a single-blind manner (patients did not know which drugs they received), since verapamil is transparent and thiocolchicine is yellow. The investigator which saw patients before and after treatment was also blinded to drug each patient had received.

Each patient received seven injections. Prior penile block with 2% lidocaine without vasoconstrictor was performed.

Patients with penile trauma, who had suffered from PD for longer than 18 months, had been operated on for PD, were on medical treatment or had discontinued medication for PD for less than three months, who were on colchicine for rheumatologic diseases (gout) or on verapamil (for hypertension) were excluded from the study.

Patients were clinically evaluated before the treatment and 30 days after the seventh injection. To assess erectile function, the simplified International Index of Erectile Function (IIEF-5) was used.

Measurement of penile curvature was made with photographs taken in three positions after a drug-induced erection (by injecting 0.3mg of phentolamine and 3μg of prostaglandin), before treatment and thirty days after completing seven injections. The greatest angle of the shaft was measured in the photographs using a protractor.

Penile ultrasound using a 7MHz transducer was performed after drug-induced erection to check the size of the plaque. The method consisted of measuring the plaque along its longest axis, before the first injection and 30 days later.

After seven applications, the investigator asked the patient if the treatment had improved the curvature and his sexual intercourse (subjective improvement).

The adverse effects of thiocolchicine were also investigated.

The studied was reviewed and approved by the Research Ethics Committee of our institution.

The statistical analysis was done using the Statistical Package for Social Sciences (IBM), version 21.0. The statistical tests used included Mann-Whitney, Likelihood Ratio Analysis and Wilcoxon test, to verify the differences between the variables.

It was adopted the significance level of 5%.

## RESULTS

Twenty-five patients were randomized; in that, 13 received thiocolchicine and 12, verapamil. Both groups were statistically similar, as shown on Table-1.

**Table 1:** Clinical characteristics of PD patients treated with verapamil or thiocolchicine.

Variable	Group	n	Mean	Standard deviation	Minimum	Maximum	25th Percentile	50th Percentile (Median)	75th Percentile	P value
AGE years	Thiocolchicine	13	55.39	9.52	39.00	69.00	48.50	56.00	63.00	0.567
	Verapamil	12	58.33	9.68	45.00	79.00	49.00	58.50	64.25	
	**Total**	**25**	**56.80**	**9.51**	**39.00**	**79.00**	**49.50**	**58.00**	**63.50**	
Stable phase months	Thiocolchicine	13	5.92	4.15	0.00	12.00	2.00	6.00	9.50	0.379
	Verapamil	12	5.00	3.81	0.00	15.00	3.25	5.00	6.00	
	**Total**	**25**	**5.48**	**3.94**	**0.00**	**15.00**	**2.50**	**6.00**	**7.00**	
Onset of plaque months	Thiocolchicine	13	9.46	4.14	2.00	15.00	6.00	9.00	13.00	0.547
	Verapamil	12	10.33	4.94	4.00	16.00	5.50	10.00	15.00	
	**Total**	**25**	**9.88**	**4.47**	**2.00**	**16.00**	**6.00**	**9.00**	**14.50**	

The mean curvature was 46.7° before the first injection and 36.2° after seven injections of thiocolchicine (p=0.019) (Table-2). In the verapamil group, the mean curvature was 50.4° before the seven injections and 42.08° after treatment (p=0.012) (Table-2). The curvature improved in 69% of the cases treated with thiocolchicine (Table-2) and in 66% of those who received verapamil, and remained stable in 23% and 33%, respectively (Table-2).

**Table 2:** Curvature and IIEF before and after tiocolchicine or verapamil injections in PD patients.

Pair of variables	N	Mean (grades)	Std. deviation	Minimum (grades)	Maximum (grades)	P value
Initial curvature (T)	13	46.77	16.48	25.00	90.00	0.019
Final curvature (T)	13	36.23	15.78	0.00	70.00	
Initial IIEF (T)	13	16.69	11.49	0.00	30.00	0.230
Final IIEF (T)	13	20.85	8.06	0.00	30.00	
Initial curvature (V)	12	50.42	10.97	40.00	70.00	0.012
Final curvature (V)	12	42.08	13.08	25.00	60.00	
Initial IIEF (V)	12	17.50	9.06	1.00	30.00	0.582
Final IIEF (V)	12	16.25	7.51	6.00	27.00	

As to sexual function, in the thiocolchicine group, there was an increase in the IIEF-5 from 16.69 to 20.85, in average, albeit without statistical significance (p=0.23) (Table-2). In the verapamil group, the IIEF-5 score dropped with no statistical significance, from 17.50 to 16.25, in average (p=0.58) (Table-2).

Concerning the plaque, 61% of those treated with thiocolchicine had a smaller size, 15% remained stable and 23% presented enlargement. In the verapamil-treated group, 8% showed a decreased plaque size, while 83% remained stable.

No adverse effect was related to thiocolchicine.

The subjective improvement was not statistically significant in the two groups (p=0.513).

## DISCUSSION

Peyronie's Disease is still difficult to manage. Although surgical treatment has been well established, the treatment of the initial phase is still controversial and the results are not always encouraging (7).

The drugs used during the initial or active phase of the disease are not very efficacious. Since its description in 1743, by François de La Peyronie (2, 3), who oriented patients to use medicinal water from the Baiege SPA, several treatments have been used with varied rates of success (16). The following oral agents have been prescribed: vitamin E, potaba [paraaminobenzoate], procarbazine, tamoxifen, steroids, and colchicine. The use of agents injected into the plaque, such as parathormone, interferon, verapamil, cortisone, and collagenase have been described (16, 17).

Oral colchicine is used during the initial phase of the disease, despite not producing high rates of success (12). Our objective was to increase the tissue level of colchicine in the tunica albuginea plaque, increasing the efficacy of treatment. However, there are severe adverse effects related to the injectable use of this drug (14, 15).

Thiocolchicine or thiocochicoside, a colchicine-derivative, was the drug proposed to replace it, increasing the concentration of this product in the plaque. This could improve the effectiveness of oral treatment without the problems of injectable use.

This randomized, prospective and single-blind pilot study showed that thiocolchicine injected directly into the plaque is well tolerated and brings benefits, since it reduces the penile curvature and plaque size. It is important to point out that thiocolchicine did not prove to be inferior to verapamil in any of the criteria analyzed.

The limitation of our study was a reduced sample size, but it was a pilot study to verify if thiocolchicine could be used for this indication.

Furthermore, verapamil was taken out of the market in our country during the study, which prevented us the inclusion of new patients. The dose of verapamil used was 5mg weekly, which could be criticized because many authors use 10mg every 2 weeks (9). Also the lack of placebo arm is a limitation of the paper; but the Ethical Committee did not approve this arm in the protocol, claiming that it would not be ethical to use it.

Another limitation of the study was the use of penile ultrasound to measure the plaque, since its use is operator dependent and sometimes cannot identify non-calcified plaques (18).

Although there were limitations this study, it clearly opens the possibility of considering intraplaque-injection of thiocolchicine for treating PD in its initial phase. The use of this medication in clinical practice will depend on the results of other randomized studies with larger samples and longer follow-up.

## CONCLUSIONS

The use of thiocolchicine injected into Peyronie's disease plaque showed improvement on penile curvature and reduction of the plaques. Thiocolchicine achieved results similar to verapamil concerning penile curvature. There were no significant side effects in this study.
